# Retrospective analysis of *Tripterygium wilfordii* polyglycoside combined with angiotensin receptor blockers for the treatment of primary membranous nephropathy with sub-nephrotic proteinuria

**DOI:** 10.1080/0886022X.2021.1918555

**Published:** 2021-04-27

**Authors:** Yuanyuan Guo, Ningning Guo, Jin Wang, Ruiqiang Wang, Lin Tang

**Affiliations:** Department of Nephrology, The First Affiliated Hospital of Zhengzhou University, Zhengzhou, P.R. China

**Keywords:** Primary membranous nephropathy, *Tripterygium wilfordii* polyglycoside, *Tripterygium wilfordii* Hook F, angiotensin receptor blocker

## Abstract

**Introduction:**

Primary membranous nephropathy (PMN) is one common cause of end-stage kidney disease. There is no optimal treatment for PMN patients with sub-nephrotic proteinuria currently. Tripterygium wilfordii polyglycoside (TWG) is a widely used traditional medicine in China and has been used to treat nephropathy for decades.

**Objective:**

To investigate the effect of TWG combined with angiotensin receptor blocker (ARB) on the treatment of PMN with sub-nephrotic proteinuria.

**Methods:**

Biopsy-proven sub-nephrotic PMN patients with normal kidney function and treated with TWG combined with ARB or ARB alone were retrospectively analyzed. The primary outcome was remission rate (complete or partial remission), and the secondary outcomes included proteinuria, serum albumin levels, estimated glomerular filtration rate (eGFR), relapse rate, and adverse events.

**Results:**

The clinical trial included 55 patients. The overall remission rates for the TWG + ARB and ARB groups after 9 months of treatment were 74.3% and 35%, respectively (*p* = 0.004). Moreover, the complete remission (CR) rate for the TWG + ARB and ARB groups in the 9th month were 45.7% and 15%, respectively (*p* = 0.044). Treatment with TWG + ARB was the independent predictor of complete remission of proteinuria (*p* = 0.048). Besides, the remission rate was higher in the TWG + ARB group than in the ARB group among patients who were positive for anti-phospholipase A2 receptor (PLA2R) antibodies (65.4% vs. 21.4%, *p* = 0.02).

**Conclusions:**

These data demonstrate that TWG may be a promising treatment for PMN patients with sub-nephrotic proteinuria, whether anti-PLA2R antibody is positive or negative.

## Introduction

Primary membranous nephropathy (PMN) remains one of the most common glomerular diseases and is characterized by subepithelial glomerular immune complexes [1]. Immunosuppressive therapies, such as administration of corticosteroids, cyclophosphamide, tacrolimus, cyclosporine, and rituximab, have been shown to increase the rate of remission and reduce progress to end-stage renal disease [2]. However, considering the possible negative side effects, such as infection, hyperglycemia, myelosuppression, and renal toxicity, immunosuppressive treatments are strictly limited to high-risk patients with nephrotic syndrome [2]. Nonetheless, recent studies have reported that approximately 60% of patients who presented with subnephrotic-range proteinuria developed nephrotic-range proteinuria during follow-up and then progressed rapidly despite the use of angiotensin-converting enzyme inhibitors (ACEi) and/or angiotensin receptor blockers (ARB) [3,4]. To date, clinical management of these patients depends on a watch and wait approach because of the inability of nephrologists to discriminate between progressive and nonprogressive disease. Thus, there is an urgent need to explore nontoxic therapies for subnephrotic membranous nephropathy patients.

Tripterygium wilfordii polyglycoside (TWG) is an extract from the Chinese medicinal plant Tripterygium wilfordii Hook F, which is a member of the Celastraceae family of perennial vine-like plants [5]. TWG has both anti-inflammatory and immunosuppressive effect and has been extensively used for centuries in China for the treatment of autoimmune diseases, including rheumatoid arthritis and systemic lupus erythematosus [5]. Besides, TWG is inexpensive and available as a tablet. Numerous Chinese patients with immunoglobulin A nephropathy (IgAN) and Henoch-Schonlein purpura nephritis have benefited from TWG since Leishi first proposed the usage of TWG in 1977 [6,7]. More encouragingly, in recent years, studies have shown that TWG combined with corticosteroid or tacrolimus therapy was effective for patients with nephrotic PMN [8,9].

Therefore, considering the negative side effects of corticosteroid and tacrolimus treatment, we performed this retrospective study to investigate the add-on benefit of TWG in treating subnephrotic PMN on angiotensin receptor blocker therapy.

## Methods

### Patients

#### Patients

This study was a retrospective analysis. Sixty-five sub-nephrotic PMN patients treated with TWG combined with ARB or ARB alone at the First Affiliated Hospital of Zhengzhou University (China) from January 2018 to May 2020 were incorporated into this trial. All the patients were diagnosed with primary membranous nephropathy according to renal biopsy and laboratory analysis for subnephrotic proteinuria (>1.0 g/day to <3.5 g/day) accompanied by a serum albumin level of <35 g/L. Patients with serum creatinine levels >115 μmol/L, active infection, diabetes mellitus, autoimmune disease, tumors, hepatitis B or C viral infection, or abnormalities in liver function tests were excluded. Patients younger than 18 years or older than 65 years of age were excluded. Patients were treated with TWG combined with ARB or ARB only. In the TWG + ARB group, 20 mg of TWG was administered three times a day (total of 60 mg per day) for more than 6 months. After 6 to 9 months, the TWG was reduced gradually by clinicians based on its therapeutic effect and patient tolerance for over 3 months. TWG was produced by Zhejiang DND Pharmaceutical Co., Ltd (Zhejiang Province, China). Different brands of ARB drugs were used from one to two tablets per day (losartan, valsartan, telmisartan). Three kinds of ARB drugs were used in two groups with no bias. The treatment time was at least 9 months. All procedures were performed following the ethical standards of the Clinical Research Committee of the First Affiliated Hospital of Zhengzhou University (2019-KY-015) and with the 1964 Declaration of Helsinki and its later amendments. Informed consent was obtained from each participant included in this study.

#### Data collection

Data on demographics, laboratory test results, renal pathology, and side effects were collected from the patients’ medical records. The symptoms and blood pressure of the patients were recorded at every visit. The monthly laboratory tests included serum creatinine level measurement, estimated glomerular filtration rate (eGFR) measurement, serum albumin level measurement, alanine aminotransferase level measurement, complete blood count, 24-h urinary protein level measurement, and tests using antibodies against m-type phospholipase A2 receptor (anti-PLA2R). All patients were followed up for >9 months.

#### Outcome measures

The primary outcome measures were the complete remission (CR) and/or partial remission (PR) of proteinuria at months 1, 3, 6, and 9. The secondary outcomes included changes in serum albumin levels, daily urinary protein levels, eGFR, and adverse effects. Urinary protein at <0.3 g/day with normal serum albumin levels and renal function was defined as complete remission (CR). A decrease in urinary protein of at least 50% from its peak value together with stable renal function and a normal serum albumin level was defined as partial remission (PR). No response was defined as cannot reach CR and PR. Relapse was defined as an increase in urinary protein levels of >1 g/day after a CR or >50% in patients who had previously reached PR.

### Statistical analysis

Continuous variables are presented as means ± SD. Non-normally distributed data are presented as the median with interquartile range and are analyzed using the Mann–Whitney U-test. Differences between the two groups were analyzed using an independent *t*-test. Analysis of variance for repeated measures before and after treatment was performed to compare the outcomes between the two groups for change over time. Multivariable analysis was performed with a Cox regression model to test the independent prognostic value of the following baseline covariates: gender, age, anti-PLA2R, and treatment. We used the chi-square test or Fisher’s exact test to compare the percentage of CR or PR in the two groups. The analysis of the probability of overall remission was performed with Kaplan–Meier curves, and differences were estimated by the log-rank test. A *P* value less than 0.05 was considered statistically significant. All statistical analyses were performed with SPSS software, version 21.0 (IBM Corp, Armonk, New York, USA).

## Results

**Figure 1.**
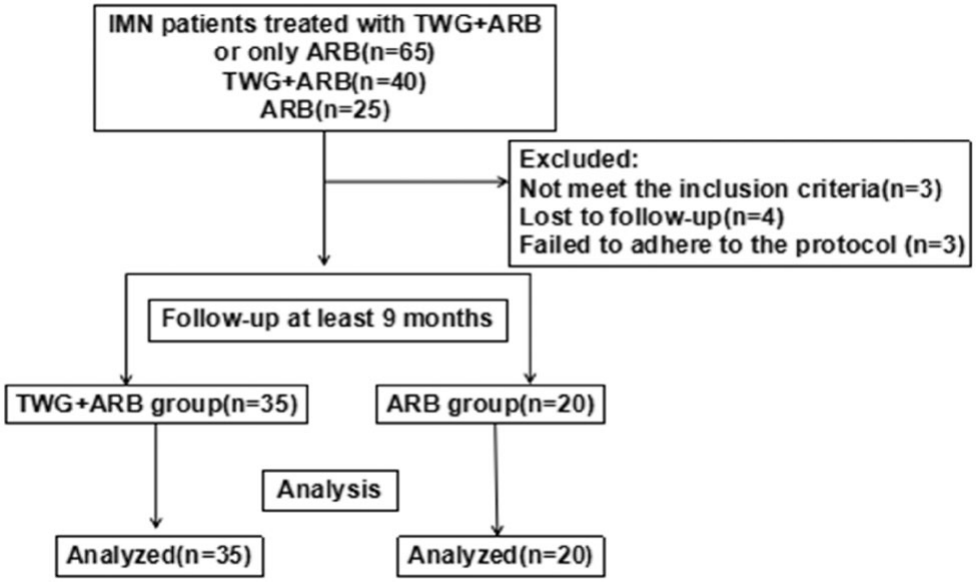
Flow diagram of patient enrollment. TWG: *Tripterygium wilfordii* polyglycoside; ARB: angiotensin receptor blocker.

From January 2018 to May 2020, 65 patients with biopsy-proven sub-nephrotic PMN were included in our study. Forty of these patients were treated with TWG combined with ARB and 25 received ARB alone. Of these 65 patients, 55 completed the observational phase (minimum of 9 months) and were enrolled in this study, with 35 in the TWG + ARB group and 20 in the ARB group. Reasons for exclusion included being over 65 years of age, unavailable for follow-up, and not following the prescription. The flowchart for patient screening is shown in Figure 1. There were no significant differences in the baseline demographic, biochemical, or histopathological characteristics in all patients and anti-PLA2R positive patients between the two groups (Tables 1 and 2).

**Table 1.** Baseline characteristics of patients.

Variable	Normal ranges	TWG + ARB	ARB	*p* Value
No. patients		35	20	
Age (yr)		49.89 ± 9.20	44.47 ± 12.23	0.118
Gender (M/F)		20/15	11/9	
Blood pressure (mmHg)				
Systolic pressure		126.74 ± 12.52	126.76 ± 8.96	0.995
Diastolic pressure		81.80 ± 8.66	78.71 ± 6.76	0.202
Hb (g/L)	130–175	131.74 ± 18.16	131.12 ± 8.31	0.865
TG (mmol/L)	<1.7	2.21 ± 1.22	1.80 ± 0.55	0.096
TC (mmol/L)	<5.2	6.86 ± 1.84	6.70 ± 1.82	0.081
SAlb (g/L)	35–55	26.89 ± 5.82	26.79 ± 4.88	0.951
Scr (μmol/L)	20–115	64.29 ± 14.25	58.47 ± 13.21	0.164
BUN (μmol/L)	2.2–8.2	4.55 ± 1.19	4.53 ± 0.78	0.936
UA (μmol/L)	200–440	290.74 ± 66.96	325.24 ± 67.44	0.088
ALT (U/L)	0–40	23.26 ± 17.17	17.47 ± 7.04	0.092
AST (U/L)	0–40	24.46 ± 8.99	21.76 ± 4.41	0.153
eGFR (mL/min/1.73m^2^)		101.42 ± 19.14	109.82 ± 15.57	0.122
Histology grading of IMN				
Stage I		5	6	
Stage I–II		9	4	
Stage II		14	7	
Stage II–III		5	2	
Stage III		2	1	
Urine protein (g/24hr)	0–0.15	2.59 ± 0.46	2.30 ± 0.34	0.307
Anti-PLA_2_R (RU/ml)	0–14	53 (13.70,86.70)	58.55 (10.8,101.6)	0.746

Values are means ± SD, medians (interquartile range), or number of patients; Hb: Hemoglobin; TG: Triglyceride; TC: Total cholesterol; ALT: alanine transaminase; AST: aspartate aminotransferase; Scr: serum creatinine; BUN: blood urea nitrogen; eGFR: estimated glomerular filtration rate; PMN: primary membranous nephropathy; TWG: tripterygium wilfordii polyglycoside; ARB: Angiotensin Receptor Blockers; Anti-PLA_2_R: antibody against m-type phospholipase A2 receptor. As non-normally distributed data, anti-PLA_2_R are presented as the median with interquartile range and are analyzed using the Mann-Whitney U test.

**Table 2.** Baseline Characteristics of anti-PLA2R-positive patients.

Variable	Normal ranges	TWG + ARB	ARB	*p* Value
No. patients		26	14	
Age (yr)		49.0 ± 9.13	44.79 ± 11.29	0.208
Gender (M/F)		16/10	9/5	
Blood pressure (mmHg)				
Systolic pressure		129.92 ± 12.06	127.5 ± 9.17	0.516
Diastolic pressure		83.04 ± 8.37	78.57 ± 8.11	0.112
Hb (g/L)	130–175	135.31 ± 16.72	131.21 ± 7.85	0.424
TG (mmol/L)	<1.7	2.34 ± 1.34	1.84 ± 0.58	0.105
TC (mmol/L)	<5.2	7.15 ± 1.90	7.01 ± 0.95	0.797
SAlb (g/L)	35–55	27.06 ± 6.16	27.89 ± 3.77	0.651
Scr (μmol/L)	20–115	65.73 ± 15.54	61.71 ± 13.91	0.424
BUN (μmol/L)	2.2–8.2	4.79 ± 1.23	4.39 ± 0.67	0.189
UA (μmol/L)	200–440	299.42 ± 64.16	314.14 ± 63.39	0.491
ALT (U/L)	0–40	21.92 ± 17.22	17.50 ± 7.29	0.367
AST (U/L)	0–40	23.65 ± 8.48	22.29 ± 3.73	0.571
eGFR (mL/min/1.73m^2^)		100.72 ± 21.47	110.74 ± 15.33	0.131
Histology grading of IMN				
Stage I		3	6	
Stage I-II		7	2	
Stage II		11	4	
Stage II-III		4	2	
Stage III		1	0	
Urine protein (g/24hr)	0–0.15	2.49 ± 0.91	2.21 ± 0.85	0.353
Anti-PLA_2_R (RU/ml) (+)	0–14	73.65 (37.18,123.50)	61.38 (55.08,157.68)	0.834

### Efficacy

**Figure 2.**
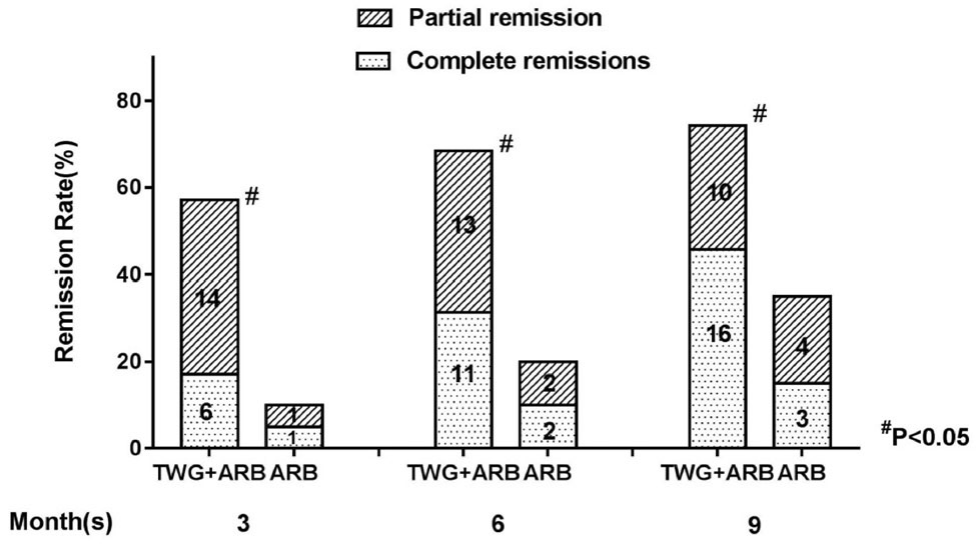
Percentage of complete and partial remissions in the TWG + ARB and ARB therapy groups. ^#^shows that the remission rate was significantly greater in the TWG + ARB group compared to the ARB group during the treatment (*p* < 0.05). TWG: *Tripterygium wilfordii* polyglycoside; ARB, angiotensin receptor blocker.

**Figure 3.**
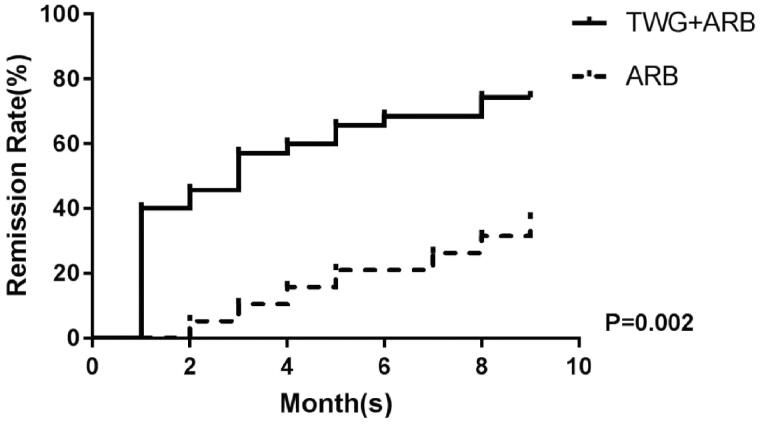
Kaplan-Meier curves for probability of overall remission for the TWG + ARB and ARB therapy groups. TWG: *Tripterygium wilfordii* polyglycoside; ARB: angiotensin receptor blocker; PLA2R, phospholipase A2 receptor.

**Figure 4.**
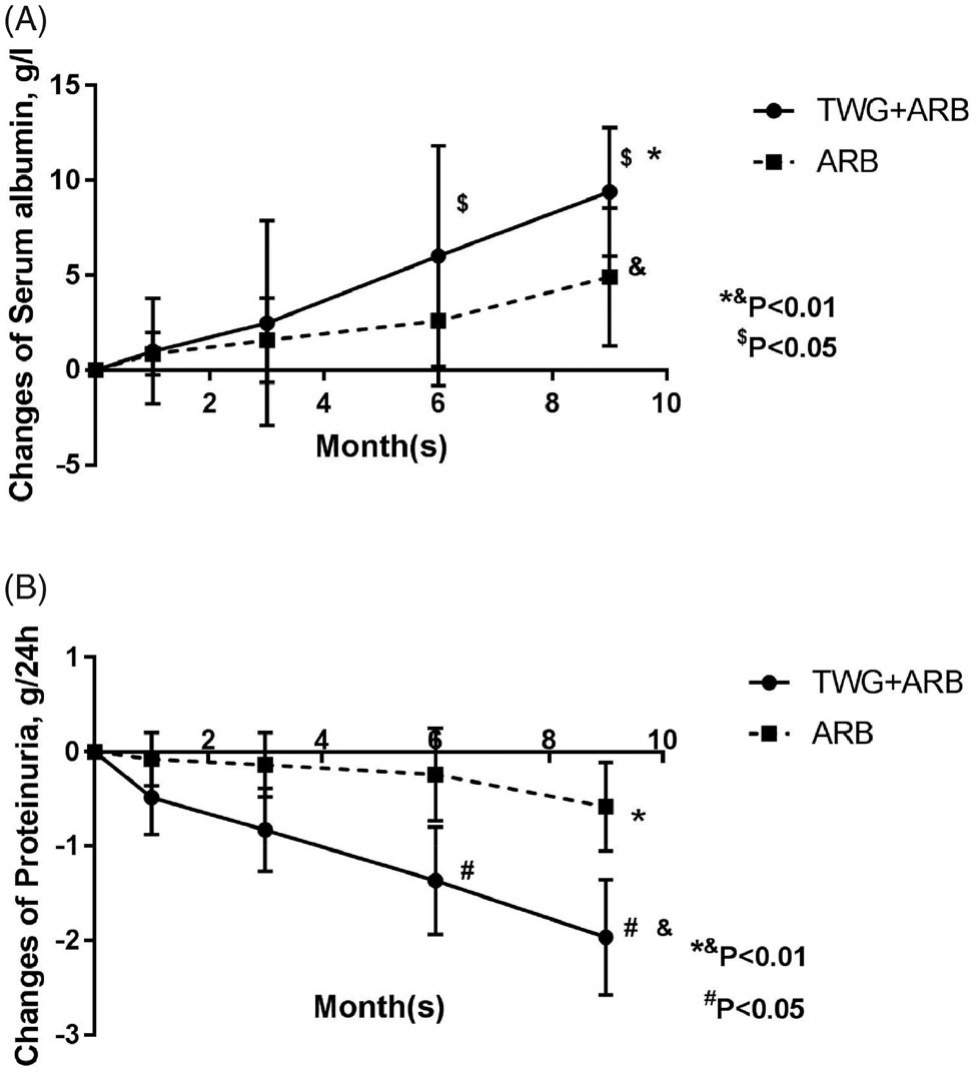
The changes of serum albumin (A) and proteinuria (B) in the TWG + ARB and ARB therapy groups. (A)*^&^statistically a significant elevation of serum albumin between the end of treatment and baseline in the TWG + ARB group and ARB group (*P* < 0.01); ^$^comparing to ARB group, the TWG + ARB group showed more obvious elevation of albumin level in 6 months and 9 months (*p* = 0.021, *p* = 0.001). TWG: *Tripterygium wilfordii* polyglycoside; ARB: angiotensin receptor blocker. (B)*^&^statistically a significant reduction of proteinuria between the end of treatment and baseline in the TWG + ARB group and ARB group (*p* < 0.01); ^#^The reduction of daily proteinuria after 6 and 9 months of treatment were more obvious in the TWG + ARB group (*p* = 0.008, *p* = 0.001). TWG: *Tripterygium wilfordii* polyglycoside; ARB: angiotensin receptor blocker

**Figure 5.**
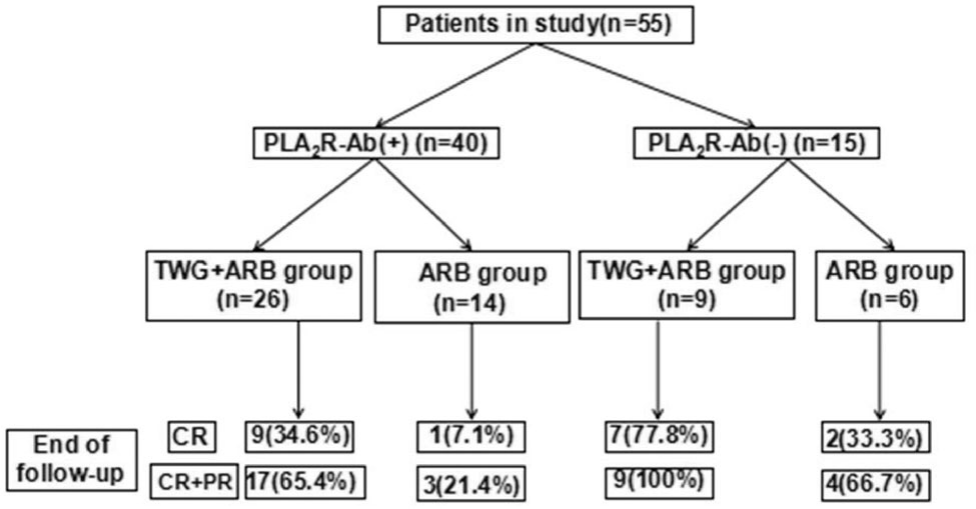
Responses of patients who were positive or negative for anti-PLA2R antibodies during follow-up. TWG: *Tripterygium wilfordii* polyglycoside; ARB: angiotensin receptor blocker; PLA2R: phospholipase A2 receptor.

**Figure 6.**
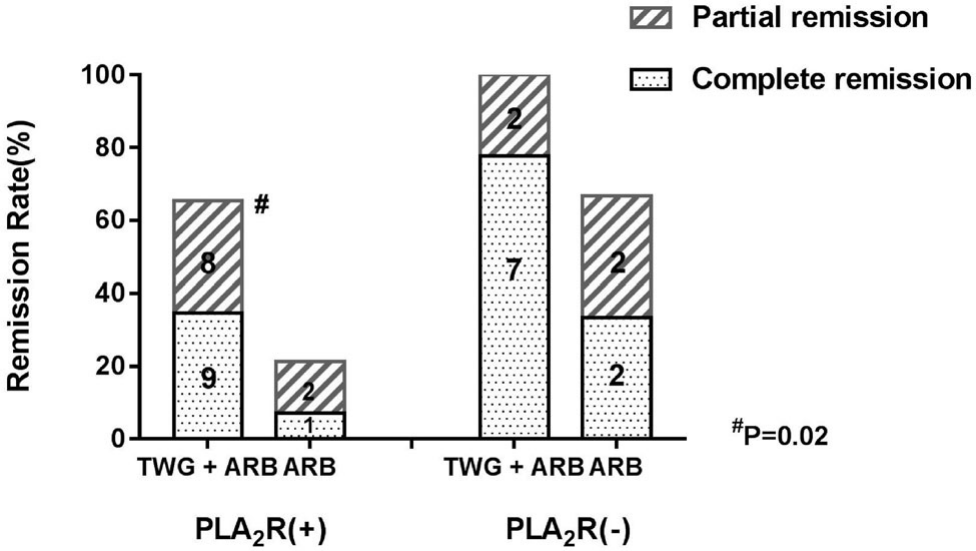
Responses of patients who were positive or negative for anti-PLA2R antibodies during follow-up. ^#^shows that the remission rate was significantly greater in the TWG + ARB group compared to the ARB group among patients with anti-PLA2R positive (*P* < 0.05). TWG: *Tripterygium wilfordii* polyglycoside; ARB: angiotensin receptor blocker

**Figure 7.**
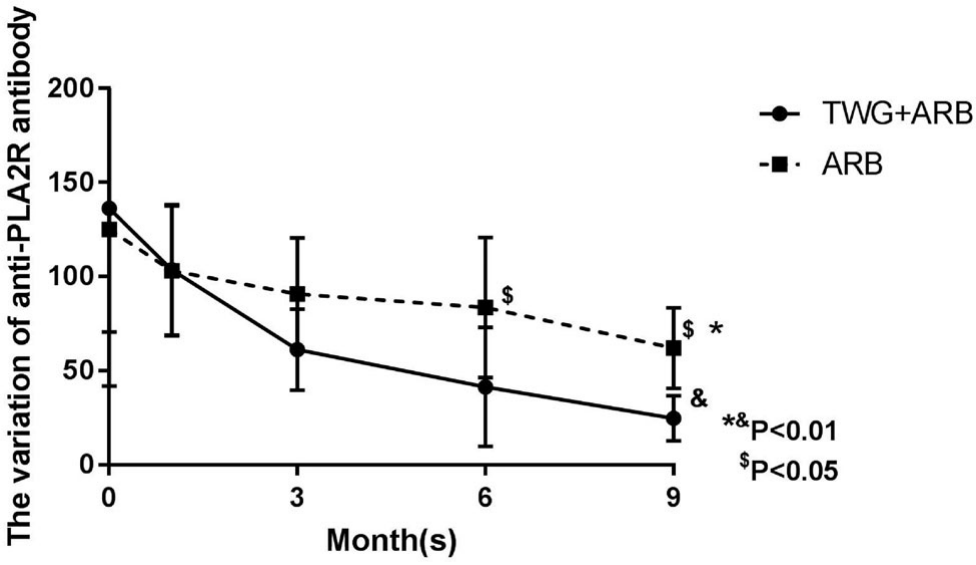
The variation of anti-PLA2R level in the TWG + ARB and ARB therapy groups. *^&^statistically a significant reduction of anti-PLA2R level between the start and end of treatment in the TWG + ARB group and ARB group (*p* < 0.01); ^$^comparing to ARB group, the mean anti-PLA2R levels after 6 and 9 months of treatment were significantly lower in the TWG + ARB group (*p* = 0.016, *p* = 0.005). TWG: *Tripterygium wilfordii* polyglycoside; ARB: angiotensin receptor blocker.

Compared with the ARB group, the TWG + ARB group had significantly higher remission rates after 6 and 9 months (Table 3, Figure 2). After 6 and 9 months, the probabilities of CR in the TWG + ARB group vs. the ARB group were 31.4% vs. 10% (*p* = 0.142), 45.7% vs. 15% (*p* = 0.044), respectively. The percentages of remission (either CR or PR) (Table 3 and Figure 2) in the TWG + ARB group vs. the ARB group by 3 months, 6 months, and 9 months were 57.1% vs. 10% (*p* = 0.001), 68.6% vs. 20% (*p* = 0.001) and 74.3 vs. 35% (*p* = 0.004), respectively. The probability of overall remission (CR + PR) was higher in the TWG + ARB group than in the ARB group during the 9 months as estimated by the Kaplan–Meier method (*p* = 0.002 by log-rank test) (Figure 3). During the 9 months of treatment, the mean levels of albumin increased from 26.89 ± 5.82 g/L to 36.27 ± 3.39g/L in the TWG + ARB group (*p* < 0.01) and from 26.79 ± 4.88 g/L to 31.69 ± 4.64 g/L in the ARB group (*p* < 0.01) (Table 4). And comparing to ARB group, the TWG + ARB group showed more obvious elevation of albumin level in 6 months and 9 months (*p* = 0.021, *p* = 0.001) (Figure 4). In addition, urinary protein excretion decreased from 2.59 ± 0.46 g/24 h to 0.57 ± 0.61 g/24 h (*p* < 0.01) in the TWG + ARB group, and from 2.38 ± 0.64 g/24 h to 1.80 ± 0.67 g/24 h (*p* < 0.01) in the ARB group by 9 months (Table 4). The reduction of daily proteinuria after 6 and 9 months of treatment were more obvious in the TWG + ARB group (*p* = 0.008, *p* = 0.001) (Figure 4). Renal function remained stable in both treatment groups, and there were no changes in eGFR (Table 4). Cox multivariable survival analysis demonstrated that treatment with TWG + ARB was the independent predictor of complete remission of proteinuria. No other influence was found for gender, age, and baseline anti-PLA_2_R (Table 5). Besides, 72.7% of PMN patients were positive for anti-PLA2R antibodies. As shown in Figures 5 and 6, among patients with anti-PLA2R positive, the percent of remission in the TWG + ARB and ARB groups was 65.4% and 21.4%, respectively, after 9 months of treatment (*p* < 0.05). Among patients who were negative for anti-PLA2R, the percent of remission in the TWG + ARB and ARB groups was 100% and 66.6%, respectively, after 9 months of therapy. During the 9 months of treatment, the mean levels of anti-PLA2R decreased in both groups (*p* < 0.01) (Figure 7). Comparing to ARB group, the mean anti-PLA2R levels after 6 and 9 months of treatment were significantly lower in the TWG + ARB group (*p* = 0.016, *p* = 0.005) (Figure 7).

**Table 3.** Remission rates and *p* value of the TWG + ARB group compared to ARB group.

Group	Month	TWG + ARB (*n* = 35)	ARB(*n* = 20)	*p* Value
CR + PR	3th	57.1% (20)	10% (2)	0.001
	6th	68.6% (24)	20% (4)	0.001
	9th	74.3% (26)	35% (7)	0.004
CR	3th	17.1% (6)	5% (1)	0.379
	6th	31.4% (11)	10% (2)	0.142
	9th	45.7% (16)	15% (3)	0.044

CR: complete remission; PR: partial remission.

**Table 4.** Laboratory parameters during the treatment of the two groups.

Variables	TWG + ARB (*n* = 35)	ARB (*n* = 20)	*p* Value
Proteinuria, g/24h			
Baseline	2.59 ± 0.46	2.38 ± 0.64	0.497
1mo	2.06 ± 0.59	2.30 ± 0.58	0.520
3mo	1.71 ± 0.44	2.24 ± 0.54	0.110
6mo	1.17 ± 0.57	2.14 ± 0.59	0.005
9mo	0.57 ± 0.61	1.80 ± 0.67	0.001
Serum albumin level, g/l			
Baseline	26.89 ± 5.82	26.79 ± 4.88	0.951
1mo	27.88 ± 4.78	27.64 ± 5.13	0.867
3mo	29.35 ± 5.40	28.36 ± 5.22	0.509
6mo	32.89 ± 5.80	29.38 ± 5.41	0.037
9mo	36.27 ± 3.39	31.69 ± 4.64	0.001
eGFR (mL/min/1.73m^2^)			
Baseline	101.42 ± 19.14	109.82 ± 15.57	0.122
1mo	99.83 ± 18.72	108.99 ± 13.93	0.163
3mo	97.96 ± 19.63	98.36 ± 12.34	0.213
6mo	100.23 ± 17.96	101.49 ± 19.37	0.121
9mo	98.36 ± 19.21	104.37 ± 18.33	0.415

**Table 5.** Predictor variables related to survival to the outcome of progression of renal disease (assessed as complete remission) at the multivariable Cox regression analysis.

Parameter	*p* Value	HR	95% CI
Age	0.880	0.914	0.285,2.932
Gender	0.578	0.774	0.313,1.911
Anti-PLA_2_R at baseline	0.066	3.979	0.913,7.337
Treatment group (TWG + ARB vs ARB)	0.048	0.274	0.0760,0.991

CI, confidence intervals; HR, hazard ratio.

### Relapse rate

Compared to the ARB group, the TWG + ARB group had a lower recurrence rate. Among the patients who experienced remission, two of 26 TWG + ARB patients (7.6%) and two of seven ARB patients (28.6%) relapsed within the treatment period (TWG + ARB group vs. ARB group, *p* = 0.001).

### Adverse effects

Among the 55 patients in this study, eight adverse events were recorded (5 in the TWG + ARB group and 3 in the ARB group). An overview of reported side effects in both groups is listed in Table 6. These side effects included liver dysfunction (3 in the TWG + ARB group, 1 in the ARB group), irregular menstruation (2 in the TWG + ARB group), and hypotension (2 in the ARB group). However, the observed side effects were mild and transitory. The liver function returned to normal after the use of a hepatic protectant, and menstruation returned to normal after TWG treatment was discontinued. No infection or bone marrow suppression occurred in either group.

**Table 6.** Adverse events.

Adverse effects	TWG + ARB (*n* = 35)	ARB (*n* = 20)
Liver dysfunction	3 (8.6%)	1 (5%)
Hypotension	0	2 (10%)
Leukopenia	0	0
Anemia	0	0
Irregular menstruation	2 (5.8%)	0

## Discussion

Membranous nephropathy (MN) is a disease characterized by the deposition of IgG and complement components onto the subepithelial layer [10]. It was first described as a specific disease entity in 1957 by David Jones [11]. MN is the leading cause of nephrotic syndrome in adults, and its incidence is approximately 1.2 per 100000 persons per year [12]. In 70–80% of these patients, MN occurs in the absence of identifiable causes and is therefore called primary MN. PMN is an autoimmune disease. The pathogenesis of PMN includes the formation of immune complexes, activation of the complement system, and subsequent release of inflammatory cytokines from the podocytes [13]. By periodic acid-Schiff (PAS) stain MN is characterized by thickening of glomerular basement membranes (GBMs) and rigid capillary lumina. In past years, patients with MN and sub-nephrotic proteinuria were considered to have a good prognosis with supportive therapy. However, several studies have shown that patients with sub-nephrotic-range proteinuria will develop nephrotic-range proteinuria and then progress rapidly [3]. In 2009, Michelle A. et al. reported that almost 60% of patients who present with low-level proteinuria subsequently develop nephrotic range proteinuria and then follow a course similar to the classic nephrotic-at-presentation group [3]. At the same time, they found that the introduction of ACEI or ARB therapy cannot alter the incidence of disease progression [3]. Elion Hoxha et al. got the same result and found that PLA2R-Ab levels are associated with a higher-risk of development of nephrotic-range proteinuria [4]. However, up to now, there has been no optimal treatment for PMN patients with low-level proteinuria who may experience an unfavorable outcome due to the extensive and severe side effects of immunosuppressive regimens [14].

TWG is a traditional Chinese medicine that has potent immunosuppressive and anti-inflammatory effects [15]. The active component of TWG is triptolide, which has been shown to inhibit lymphocyte proliferation, block NF-κ B activation and regulate inflammatory reactions mediated by toll-like receptors [5]. These characteristics help to explain the powerful therapeutic effects of TWG on many autoimmune diseases. Furthermore, TWG has been widely and successfully used in China for the treatment of IgAN and purpura nephritis for over 40 years [6,7,16,17], and multiple clinical studies reported that TWG has beneficial therapeutic effects on the remission of idiopathic refractory nephrotic syndrome [18]. Studies have shown that TWG displayed a protective effect against renal fibrosis by decreasing levels of inflammatory mediators and, thereby, protecting the glomerular filtration barrier and podocytes from injury [5,14,19]. Currently, some studies on the treatment of membranous nephropathy by TWG are underway. Chen et al. reported that triptolide reduced proteinuria in experimental membranous nephropathy and protected against C5b-9-induced podocyte injury *in vitro* [20]. In addition, Zhou et al. found that triptolide attenuated the inflammatory response in membranous glomerulonephritis in a rat model *via* downregulation of the NF-κB signaling pathway [21]. Shang et al. conducted a retrospective study showed that TWG combined with tacrolimus resulted in a higher remission rate (85.7%) and lower relapse rate for treating PMN than tacrolimus combined with methylprednisolone group [9]. Furthermore, a randomized control trial reported that the remission rate is 86.9% in the TWG combined with prednisone group, similar to the tacrolimus combined with prednisone group, in treating PMN [8]. TWG is sometimes used in our kidney center to treat PMN and some clinical trials are being planned. In the present study, we evaluated the efficacy and safety of TWG combined with ARB in patients with sub-nephrotic PMN. The overall remission rates for the TWG + ARB and ARB groups after 9 months of treatment were 74.3% and 35%, respectively (*p* = 0.004), showing TWG as an effective medicine for PMN. Furthermore, we report few side effects with this therapy, suggesting that oral TWG is a safe treatment.

Circulating autoantibodies against PLA2R were detected in approximately 70% of patients with MN, and PLA2R antibody levels correlated with disease severity and outcomes [22,23]. Hoxha et al. found that anti-PLA2R antibody levels were associated with a higher risk of developing nephrotic-range proteinuria [4]. Furthermore, a decrease in the PLA2R antibody level predicts the remission of proteinuria. In addition, studies indicated that the PLA2R titer was associated with progressive loss of kidney function and a high risk for relapse [24,25]. According to the Kidney Disease: Improving Global Outcomes organization (KDIGO.org), the PLA2R antibody was listed as a high-risk factor resulting in the progression to end-stage kidney disease. Thus, appropriate treatment strategies are required for PLA2R antibody-positive patients. In our study, the remission rate was significantly higher in the TWG + ARB group compared to the ARB group (65.4% vs. 21.4%, *p* = 0.02), indicating that TWG may be a particularly effective and safe medicine for the treatment of PMN patients who are anti-PLA2R positive.

In other studies, the TWG regimen produced some side effects, such as the upset stomach, liver injury, reversible infertility in men, and amenorrhea in women [26,27]. In our study, we observed liver dysfunction and amenorrhea in several patients which agree with previous studies of TWG treatment.

This study had several limitations. First, this was a retrospective, observational study, treatment assignments were not protocol-driven and clinicians decided which treatment to use based on their experience, which may lead to selection bias. Second, this was a single-center study of Chinese patients, therefore, the results may not be generalizable and further studies will be needed to validate the efficacy in non-Asian ethnicities. Third, the follow-up period was not long enough and the sample size was small, leading to potential bias in the evaluation of the efficacy and safety of TWG therapy. In addition, some remissions in our study were partial, and relapses of PMN and disease progression are more common in patients with PR. Therefore, we continue to follow up with the patients in this study, and randomized, controlled studies involving larger numbers of patients and longer follow-up periods should be conducted.

## Conclusion

This retrospective study concludes that TWG appears to be effective and safe for the treatment of sub-nephrotic PMN patients on top of ARB treatment. To further verify the current results, large-scale and high-quality randomized clinical trials are warranted. Long-term clinical trials will need to be conducted to ascertain the long-term efficacy and side effects of TWG on PMN patients with sub-nephrotic proteinuria.
